# *Fusobacterium* bacteremia presenting with inferior mesenteric vein thrombosis

**DOI:** 10.1002/ccr3.7617

**Published:** 2023-06-29

**Authors:** Surendra Sapkota, Suraj Shrestha, Sandipa Sharma, Subash Sapkota, Lauren A. Solis, Abhishek Kalla

**Affiliations:** ^1^ Saint Agnes Hospital Baltimore Maryland USA; ^2^ Maharajgunj Medical Campus Institute of Medicine Kathmandu Nepal; ^3^ Shivanagar Primary Health Care Center Chitwan Nepal; ^4^ Jersey City Medical Center Jersey City New Jersey USA; ^5^ Ross University School of Medicine Bridgetown Barbados

**Keywords:** Fusobacterium, Lemierre's syndrome, mesenteric vein, thrombosis

## Abstract

Isolated mesenteric vein thrombosis associated with *Fusobacterium* is rare. Physicians should be aware regarding the association of *Fusobacterium* with thrombosis at various sites.

## Graphical abstract

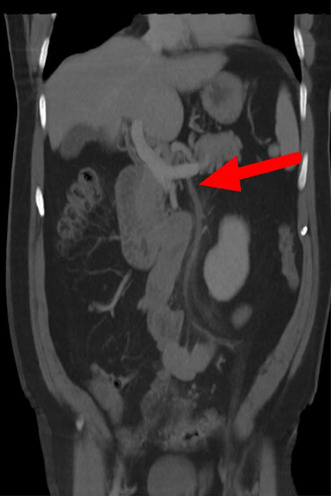

## 1 INTRODUCTION

*Fusobacterium* species are fastidious gram‐negative anaerobes generally found as commensal flora in the human oropharyngeal microbiome.^1^ The most common species isolated within this genus are *F. necrophorum* and *F. nucleatum*, the former is more commonly known to cause Lemierre's syndrome.^2^ Variants of Lemierre's syndrome caused by *Fusobacterium* species have also been described in multiple anatomic locations such as hepatic veins, ovarian veins, suprahepatic veins, and mesenteric veins.^3^, ^4^, ^5^, ^6^

Mesenteric vein thrombosis (MVT) is a rare condition that accounts for one in 5000 to 15,000 hospital admissions and one in 1000 admissions to the emergency department, with high mortality (19%–23%).^7^ Primary or idiopathic MVT accounts for 21%–49% of the cases and the proportion of patients with idiopathic cases decreases with more extensive evaluation.^8^ MVT can occur as a complication of appendicitis, cholecystitis, pancreatitis, diverticulitis, and other intra‐abdominal infections.^9^ Prothrombotic states or thrombophilia and local intra‐abdominal infections are the main causes of MVT.^10^ In a study on 22 cases of *Fusobacterium*‐related pylephlebitis, 41% involved only the portal vein, 32% involved some combination of the portal vein, superior mesenteric vein (SMV), inferior mesenteric vein (IMV), or splenic vein, and 14% involved SMV alone. There was isolated involvement of the right hepatic vein in two cases and only in one case was the IMV affected.^9^

Hereby, we report a case of a 59‐year‐old male who presented with a week of back pain and malaise, found to have an acute inferior mesenteric vein thrombosis and blood culture ultimately growing *Fusobacterium species* who responded well to medical therapy.

## 2 CASE REPORT

A 59‐year‐old Caucasian male with a 10‐year medical history of hypertension and obesity presented to the emergency department complaining of bilateral lower back pain and malaise for 1 week followed by shortness of breath of 1 day duration. The patient denied nausea, vomiting, abdominal pain, fever, flank pain, and urinary symptoms. He described back pain as moderate in intensity and achy in nature, with no alleviating or aggravating factors. He reported no surgical history nor any known history of malignancy or thrombotic disorders in the family. He denied using tobacco and illicit drugs and reported occasional alcohol consumption (approximately 6–8 units per week).

**FIGURE 1:**
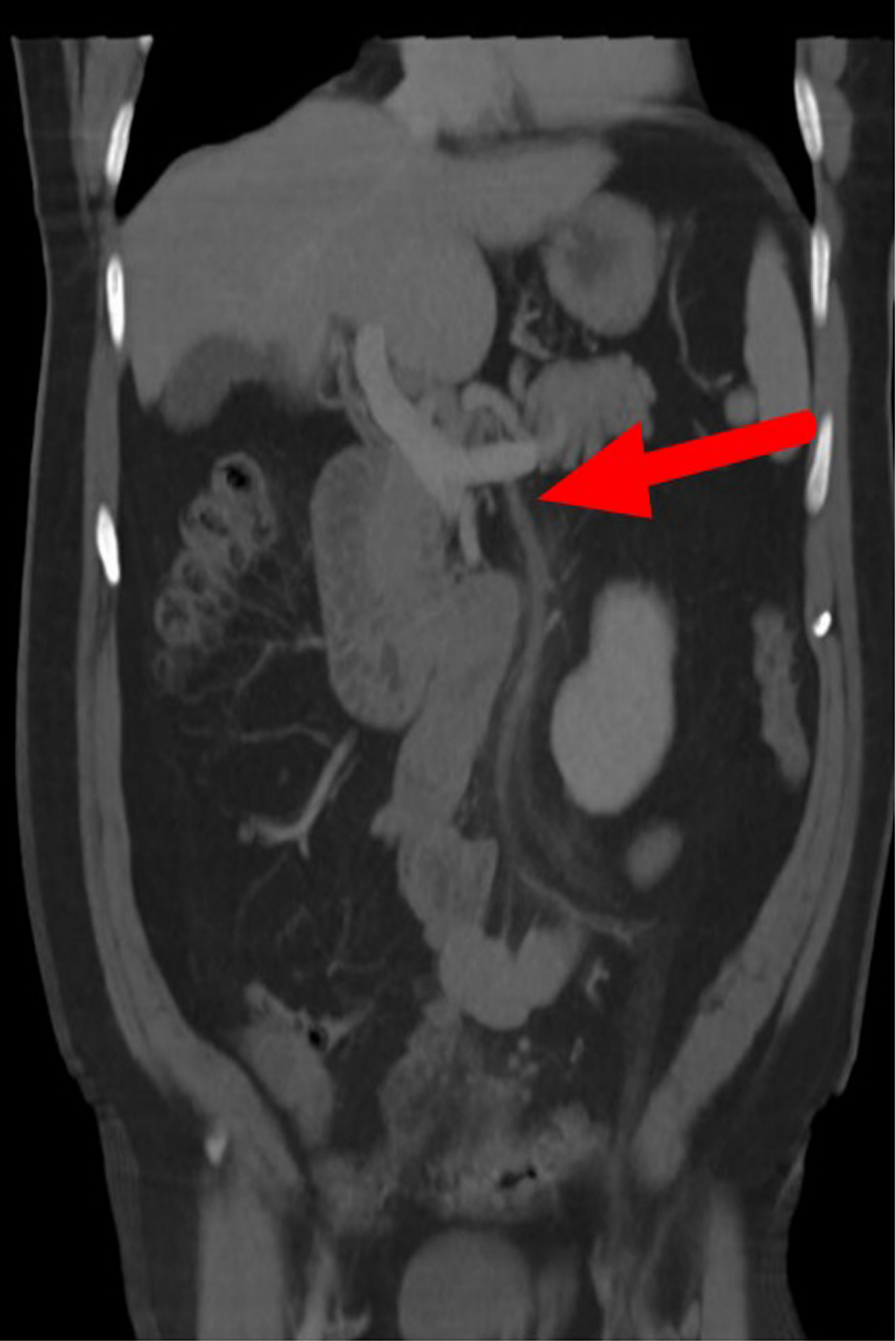
CT abdomen/pelvis 3D coronal view demonstrating acute/early subacute partially occlusive thrombosis of the inferior mesenteric vein with mild adjacent perivascular stranding.

In the emergency department, the patient was afebrile, tachycardic at 121/min, blood pressure 132/82 mm Hg, and oxygen saturation was 96% in room air. Laboratories demonstrated leukocytosis, hyperbilirubinemia, and transaminasemia. Blood cultures were sent. An abdominal ultrasound showed no ductal dilation of the gallbladder; however, the gallbladder was poorly seen. A contrast‐enhanced computerized tomography (CT) of the abdomen and pelvis demonstrated an acute/early subacute partially occlusive thrombosis of the IMV with mild adjacent stranding.(Figure 1) There was no evidence of liver abscess, diverticulitis, or colitis. Vascular surgeons were consulted, and the patient was started on apixaban 10 mg twice daily and was discharged on the same dose with a plan to transition to 5 mg twice daily after a week. The anaerobic bottle showed *Fusobacterium* and the aerobic bottle showed no growth. Emergency department providers made multiple attempts to contact the patient but were unsuccessful.

Only 2 weeks later, the patient returned to the ED with similar symptoms. The patient did not receive any antibiotics between the previous visit and second visit as the providers were unable to reach the patient via phone. In the emergency department, the patient was afebrile, tachycardic at 130/min, blood pressure of 120/84 mm Hg, and oxygen saturation was 96% in the room air. Laboratories were significant for leukocytosis and thrombocytosis. There was no evidence of urinary tract infection on urinalysis. He tested negative for the influenza virus and COVID‐19 virus. The urine drug test was negative. The chest CT showed no evidence of pulmonary emboli or acute pulmonary process. Laboratory and imaging workup on admission has been summarized in Table 1. Given the recent mesenteric thrombus, the patient was continued with apixaban 5 mg twice daily. The patient was started on intravenous piperacillin‐tazobactam per infectious disease physicians' recommendations. As the evaluation of an underlying hypercoagulable state would not alter the course of immediate treatment of the thrombus and could ultimately be affected by *Fusobacterium* bacteremia, the hematologist recommended that the workup be performed outpatiently. The medical team also felt that the thrombocytosis was likely reactive in nature, as the patient's platelet count was within normal limits at his previous visit. His clinical course improved and repeated blood cultures were negative twice. After a five‐day hospital stay, the patient was discharged with amoxicillin‐clavulanic acid (875/125 mg) twice daily for a total duration of 4 weeks and apixaban 5 mg twice daily (HASBLED score‐2), with duration to be determined by the hematologist at the outpatient follow‐up.

**TABLE 1:** Laboratory parameters with reference range.

Laboratories	First emergency visit	Hospital admission	Follow‐up	Reference ranges
White blood cells	16 K/uL (with 79.3% neutrophils)	16.1 K/uL	8.0 K/uL	4.0–11.0 K/uL
Red blood cells	5.03 M/uL	4.41 M/uL	4.90 M/uL	4.50–5.90 M/uL
Hemoglobin	15.3 g/dL	13.4 g/dL	14.7 g/dL	13.0–17.0 g/dL
Hematocrit	45%	39.7%	44.0%	41.0%–50.0%
MCV	89.5 fL	90.0 fL	89.8 fL	80.0–99.0 fL
Platelet count	246 K/uL	601 K/uL	369 K/uL	150–400 K/uL
Sodium	134 mEq/L	137 mEq/L	139 mEq/L	136–145 mEq/L
Potassium	4.5 mEq/L	4.9 mEq/L	4.3 mEq/L	3.5–5.1 mEq/L
Blood urea nitrogen	11 mg/dL	25 mg/dL	18 mg/dL	8–21 mg/dL
Creatinine	1 mg/dL	0.8 mg/dL	1.02 mg/dL	0.6–1.2 mg/dL
Total bilirubin	2.1 mg/dL	0.7 mg/dL	0.6 mg/dL	<1.5 mg/dL
AST	144	63 U/L	20 U/L	<39 U/L
ALT	214	108 U/L	24 U/L	<42 U/L
ALP	259	274 U/L	57 U/L	35–126 U/L
Prothrombin time	12 s	12.7 s	–	9.2–12.2 s
International ratio	1.1	1.2	–	0.9–1.1
aPTT		28 s	–	21–32 s
VBG	pH 7.41	–	–	–
	pCO_2_‐36 mm Hg			
	pO_2_ 63 mm Hg			
	HCO_3_ 23 mEq/L			
	Lactic acid 1.7 mmol/L			
Imaging findings during hospital admission				
USG abdomen	Hepatic steatosis, contracted gallbladder, mildly increased velocity of the main portal vein but no hydronephrosis, or other obvious infections.			
Transthoracic echocardiogram	Ejection fraction of 60%, mild enlargement of the right ventricle and bilateral atria, mild mitral regurgitation, and mild aortic regurgitation, mild tricuspid regurgitation with right ventricular systolic pressure of 40 mm Hg, and no pericardial effusion.			
Venous duplex of the bilateral lower extremities	No venous thrombosis			

Abbreviations: AST, aspartate aminotransferase; ALT, alanine aminotransferase; ALP, alkaline phosphatase; aPTT, activated partial thromboplastin time; VBG, venous blood gas.

**FIGURE 2:**
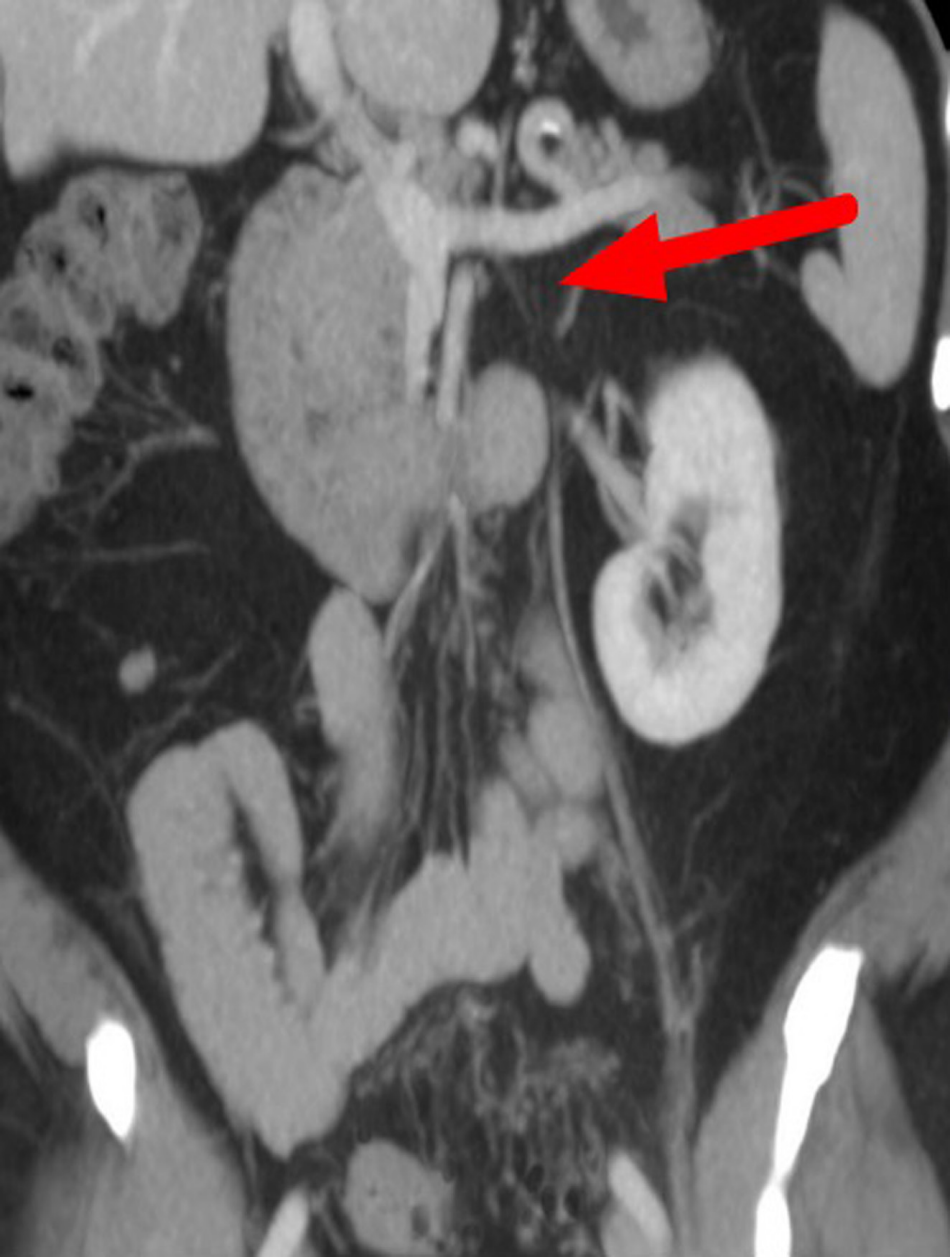
CT abdomen/pelvis 3D coronal view showing atretic appearance of the inferior mesenteric vein compared to the prior study along with resolution of previous perivascular stranding.

At the follow‐up appointments, he was doing well on antibiotics and anticoagulation. Laboratories were significant for the resolution of leukocytosis, thrombocytosis, and transaminitis. (Table 1 for laboratory work on admission and follow‐up visit) A repeat CT abdomen/pelvis 2 months after discharge revealed a diffusely atretic inferior mesenteric vein but no obvious thrombus compared to the previous scan.(Figure 2) The BCR‐ABL and JAK2 panel mutations (JAK2 V617F, JAK2 EXON 12–13, Calreticulin, MPL) were negative. A referral was provided to the gastroenterologist for a colonoscopy. The patient was explained that IMV thrombosis was likely due to the underlying *Fusobacterium* infection, making the thromboembolism a provoked one, and that he would only need 3 months of anticoagulation, which he had completed at the time of his second appointment with the hematologist. No further workup or follow‐ups for hematology were recommended.

## 3 DISCUSSION

*Fusobacterium necrophorum* is a nonspore‐forming, obligate anaerobic, gram‐negative bacillus and is unique for its ability to cause severe infection. Pylephlebitis, or suppurative thrombophlebitis of the portomesenteric venous system, is a rare complication of intra‐abdominal infections and is an exceedingly rare sequela of *Fusobacterium* spp. septicemia.^11^ The association between *F. nucleatum* and thrombosis, although less established, has been reported in several cases, including those of the iliac, portal, and mesenteric veins.^12^, ^13^, ^14^, ^15^ The spread of *Fusobacterium* to the large intestine and associated vasculature has been proposed to occur via a hematogenous route and translocation from the oral cavity to the intestine.^1^ At the molecular level, *Fusobacterium* species bind and invade various cell types and stimulate inflammatory responses.^16^ The adhesin protein, *Fusobacterium* adhesin A (FadA), binds the cadherins of colonic epithelial cells and vascular endothelial cells, facilitating adherence and invasion of these species.^17^, ^18^ F. nucleatum has also been demonstrated to induce inflammatory host cytokines, including IL‐6, IL‐8, and TNFα.^18^ These combined effects create a platform for endothelial cell dysfunction and inflammation, thus promoting thrombus formation. F. nucleatum has been shown to promote colorectal carcinogenesis through direct effects on the expression of oncogenic and inflammatory genes, as well as through suppression of host immunity.^1^ Thrombosis in atypical locations warrants consideration of a hypercoagulable state (e.g., malignancy and rheumatological conditions) and colonoscopy for colon cancer and inflammatory bowel disease.^1^, ^19^

Diagnosis of mesenteric vein thrombosis is based on confirmatory imaging findings of the portal vein or mesenteric vein thrombosis in the setting of systemic infection. Intravenous contrast‐enhanced computerized tomography (CT), ultrasonography, or magnetic resonance imaging (MRI) can all be used to establish the diagnosis, with the former reported as the modality of choice given its availability and high sensitivity.^20^ However, despite increasing evidence of the association of *Fusobacterium* with thrombosis, blood cultures are not consistently included as part of the standard evaluation.^1^ Initial evaluations including urinalysis, CT chest/abdomen/pelvis, abdominal ultrasound, and echocardiogram were unremarkable revealing no source of infection. Neck CT was considered, but was ultimately canceled because infectious disease consultants felt that the patient did not reveal signs or symptoms of oral infection with respect to Lemierre's syndrome.

The management of this entity is the timely initiation of antibiotics and anticoagulation. Antibiotic therapy with anaerobic coverage must be rapidly introduced. Mortality is difficult to estimate but can be high, up to 25%, and depends on the timing of antibiotic initiation.^21^ As penicillin‐resistant strains have been reported, empiric therapy should consist of clindamycin or metronidazole or the use of a combination of beta‐lactams with beta‐lactamase inhibitors.^22^ Unlike Lemierre's syndrome, anticoagulation is recommended for all cases of acute or subacute mesenteric vein thrombosis and remains the cornerstone of treatment.^23^ A retrospective study of 120 patients with MVT diagnosed between 2000 and 2015 by Salim et. al in 2018 concluded that immediate anticoagulation is an effective first‐line therapy in patients with MVT.^23^

Anticoagulation should be initiated as soon as the diagnosis of IMV is made, even intraoperatively or in the presence of bleeding as it has been shown to significantly improve survival.^8^ Recurrence most commonly occurs in the first 30 days after presentation. The reported rates of 0%–25% can be decreased to 0%–3% in patients who continue on anticoagulation.^24^, ^25^

Overall, there is a trend to benefit from early anticoagulation due to the risk of bowel ischemia and infarction, especially when a mesenteric branch is involved.^11^ Thrombus formation and rapid bacterial growth can cause septic embolization at distant sites, including the lungs, joints, bones, skin, and soft tissues, muscles, liver, spleen, kidneys, heart, and brain.^3^ The timely initiation of anticoagulation may reduce septic embolization of the liver from infected portal thrombi and prevent liver abscesses.^3^ In a retrospective study of MVT patients, 13 patients seen before 1995 who did not receive anticoagulants were compared with 28 patients seen after 1995 who received anticoagulants. The patients in the latter group had a shorter mean hospital stay duration (13 vs. 26 days), reduced hospital mortality (11% vs. 39%), and less need for surgery (33% vs. 85%).^26^ Choices of anticoagulation include unfractionated heparin in the acute setting, which later transitioned to low molecular weight heparin, warfarin, or direct oral anticoagulants (DOAC).^27^ Lifelong anticoagulation should be considered in IMV thrombosis cases with persistent hypercoagulable state, irreversible systemic condition, or idiopathic cases.^8^ In a case of portal vein thrombosis with underlying *Fusobacterium* pylephlebitis, warfarin anticoagulation was performed for 6 months with improved clinical outcome.^14^ Several clinicians have felt comfortable using DOAC for mesenteric vein thrombosis. Cheng et al. have reported the use of rivaroxaban for 3 months in their case of mesenteric and portal vein thrombosis with *Fusobacterium* nucleatum bacteremia.^1^ Lazar et al. have treated a case of portomesenteric thrombosis secondary to *Fusobacterium* with apixaban 5 mg twice daily for 3 months with an uneventful course and no bleeding events.^9^ Thus, in the absence of any contraindications or presumed risk, anticoagulation therapy should be considered. A duration of 3–6 months of anticoagulation is recommended for patients with reversible causes like the patient described above with an evaluation of risks and benefits. Surgical intervention is reserved for cases with persistent septic emboli, showering, or continued propagation of thrombosis.^28^

## 4 CONCLUSION

*Fusobacterium* is a clinically important yet under‐recognized contributor to venous thrombosis at many anatomic sites. Prompt antibiotic therapy, supportive care, and anticoagulation therapy with DOAC for 3 months resulted in a favorable outcome in our case. In patients with portomesenteric thrombosis, blood cultures should be consistently included as part of the standard evaluation, especially when there are vague systemic complaints such as malaise, fatigue, etc. and suspicion of underlying infection. The consequences of untreated MVT can be debilitating, including sepsis and death; therefore, our aim is to increase the awareness of physicians of *Fusobacterium*‐associated mesenteric vein thrombosis that requires immediate antibiotics and anticoagulation.

## AUTHOR CONTRIBUTIONS

**Surendra Sapkota:** Conceptualization; data curation; investigation; writing – original draft; writing – review and editing. **Suraj Shrestha:** Writing – original draft; writing – review and editing. **Sandipa Sharma:** Writing – original draft; writing – review and editing. **Subash Sapkota:** Writing – original draft; writing – review and editing. **Lauren A Solis:** Writing – original draft; writing – review and editing. **Abhishek Kalla:** Writing – original draft; writing – review and editing.

## FUNDING INFORMATION

No funding was received for this study.

## CONFLICT OF INTEREST STATEMENT

None to declare.

## ETHICS STATEMENT

Ethical approval is not required for this study in accordance with local or national guidelines.

## CONSENT

Written informed consent was obtained from the patients for publication of this case report and accompanying images. A copy of the written consent is available for review by the editor in chief of this journal on request.

## Data Availability

The data that support the findings of this study are available from the corresponding author upon reasonable request.
